# Musculoskeletal pain among offshore wind industry workers: a cross-sectional study

**DOI:** 10.1007/s00420-020-01544-3

**Published:** 2020-04-27

**Authors:** Marcial Velasco Garrido, Janika Mette, Stefanie Mache, Volker Harth, Alexandra M. Preisser

**Affiliations:** Institute for Occupational and Maritime Medicine (ZfAM), University Medical Center Hamburg-Eppendorf, Seewartenstr. 10, 20459 Hamburg, Germany

**Keywords:** Offshore, Ergonomics, Musculoskeletal pain, Physical strain

## Abstract

**Objective:**

To assess whether there are differences in musculoskeletal pain among different types of occupations offshore and their relationship to ergonomic demands.

**Methods:**

We conducted a web-based cross-sectional survey among workers from offshore wind energy companies operating within the German exclusive economic zone. We selected workers with regular offshore commitments and at least 28 days spent offshore in the past year (*n* = 268). Musculoskeletal pain was assessed using the Subjective Health Complaints inventory (SHC), which considers the past month.

**Results:**

Of the 268 male participants eligible for analysis, 54% reported back pain 50.4% neck pain, 40.3% lower back pain, 35.5% shoulder, 23.3% arm and 22.1% leg pain, all of them during the past month. Compared to other offshore occupations, technicians reported more frequently arm (OR 3.13; 95% CI 1.58–6.19), back (OR 1.97; 95% CI 1.15–3.39), shoulder (OR 1.94; 95% CI 1.11–3.40) and neck pain (OR 1.89; 95% CI 1.11–3.22). After adjusting for age and nationality, lifting and carrying heavy loads were associated with all types of pain except leg pain. Overhead work, work in awkward postures, and the use of personal protection equipment and heavy tools was associated with shoulder, back and arm pain.

**Conclusions:**

Our findings suggest that occupational health counselling, health promotion and preventive interventions of offshore wind energy workers needs to consider the specific tasks of the employee and be particularly tailored to the ergonomic needs of technicians.

## Introduction

By the year 2018, the total installed power capacity of offshore wind energy outreached 20 gigawatt (GW) (Fraunhofer Institute for Energy Economics and Energy System Technology 2019a), accounting for around 4% of worldwide wind energy capacity (Global Wind Energy Council 2019). The UK with 37% and Germany with 28% lead the share of installed offshore wind energy capacity, although this industry is rapidly growing in other countries, such as China (Fraunhofer Institute for Energy Economics and Energy System Technology 2019). The commitment of governments and industry with the installation of offshore wind technology continues to expand worldwide (US Department of Energy and US Department of the Interior 2016; Federal Ministry for Economic Affairs and Energy 2015). Accordingly, there is an increasing number of workers involved in the construction and operation of offshore wind installations. Not only is the total capacity growing, but also the dimensions of the installations. Smaller turbines with nominal capacities of 3–5 MW are being replaced by units with 7–8 MW and even more powerful units of 8–10 MW are being planned (Fraunhofer Institute for Energy Economics and Energy System Technology 2019b; Foundation Offshore Wind Energy 2019). Consequently, turbine rotor diameter and hub height have grown from approximately 70 m and 64 m, respectively, by the year 2000 to 140–150 m and 90–107 m in 2018 (Fraunhofer Institute for Energy Economics and Energy System Technology 2019b; Foundation Offshore Wind Energy 2019). These dimensions as well as the technical characteristics of the installations pose specific physical and ergonomic demands on the workforce, particularly on the technical personnel who are typically in charge of inspecting, performing routine maintenance and repairing the installations (Milligan et al. 2019). In our prior publication we described offshore wind technicians as regularly being exposed to unavoidable physical demands and ergonomic challenges such as climbing, working at extreme heights and in confined spaces, handling heavy tools and wearing bulky personal protection equipment when performing their tasks (Velasco Garrido et al. 2018a). Working at offshore wind energy installations has been proven to be a form of heavy physical work, as demonstrated by research assessing cardiac output during preparative trainings (Preisser et al. 2019).

According to an extensive review of the literature, there is reasonable evidence of a causal relationship between working in awkward postures, lifting heavy loads and heavy physical work and the development of work-related musculoskeletal disorders, which not only include musculoskeletal pain but also objectified disorders (e.g., tendinitis or carpal tunnel syndrome) (da Costa et al. 2010). A recent study using a job exposure matrix has shown consistent associations with musculoskeletal pain for physical working conditions (working with bent/twisted back, with arms over the shoulders, kneeling, lifting and carrying – among other) (Madsen et al. 2018). Musculoskeletal pain is of particular relevance since it is associated with reduced work ability independently of location for both younger and older workforce (Bayattork et al. 2019) as well as with long-term sickness leave (Andersen et al. 2011).

The aim of this paper is to investigate whether there are differences in health complaints, particularly musculoskeletal pain, between workers with expected exposure to high physical demands, such as technicians, and other workers in offshore wind energy parks.

## Methods

### Study design and population

The study was conducted in accordance with the Declaration of Helsinki, and the protocol was approved by the Ethics Review Committee of the Hamburg Medical Association.

We conducted a cross-sectional online survey. Data were collected between September 2016 and January 2017. The source population are workers of offshore wind installations located in the German exclusive economic zone of the North and Baltic Seas. At the time of the survey, 22 wind farms were either already in operation or under construction in this area (Fraunhofer Institute for Energy Economics and Energy System Technology 2019c), with estimated 5000–7600 employees having regularly or irregularly offshore commitments (Federal Ministry for Economic Affairs and Energy 2015). Due to the nature of the branch which is characterized by its high fragmentation with many companies involved, there are no other reliable data on the offshore workforce, than the mentioned estimations. To ensure that the collective had sufficient offshore experience, we restricted the sample to workers with regular offshore deployments or with a total of at least 28 days offshore during the past year if they were working on an irregular schedule. Women (*n* = 28) were also excluded from further analyses, since they differed statistically regarding relevant sociodemographic characteristics, such as marital and parental status.

### Recruitment

Participation in the survey was anonymous and voluntary. Participants were recruited by contacting occupational physicians, health and safety managers, and human resources departments of relevant companies by telephone, e-mail and regular mail. In addition, we promoted the study on relevant online platforms and forums of offshore workers and presented the study at the “Round-table Maritime Safety Partnership”, a regular meeting of key stakeholders organized by the German Offshore Wind Energy Foundation (Stiftung Offshore Windenergie 2019).

### Questionnaire

Access to the online questionnaire was possible through a URL and QR-code provided in all written information materials (leaflets, e-mails, and social-media postings) used for recruitment. The questionnaire had two versions: German and English. The first page of the questionnaire provided information on the study purpose and required participants to provide consent by ticking the corresponding box (“I hereby confirm that I have read and understood the study information and data protection policy above. I agree to participate”) prior to proceed with collection of further data. Termination of the survey was possible at any stage. The questionnaire was piloted and refined with the help of offshore workers. Completion of the questionnaire—including topics and instruments not reported in this paper—required a median time of 24 min.

### Sociodemographic variables

We collected data on gender, age, marital status (“single” or “living in a relationship”), parental status (“children under 18 years living at home” or “no children”), and nationality (“German” or “other”).

### Job characteristics

Job characteristics included offshore experience (“less than 1 year” – “1 to 3 years” – “more than 3 years”), occupation type (“technician” – “other” (including site manager, catering, room service, quality management, paramedics, etc.), offshore work schedule (“regular”, including 14/14 day rhythms as well as other models) – “occasional commitments”), work shifts (“rotating shift” – “non-rotating shift”) and project phase of the wind farm (“under construction” – “operation”).

### Physical strains

Work related exposures were assessed by asking the participants to self-assess their level of exposure to ergonomic factors and/or physical demands in a five-point Likert scale (“always” – “often” – “sometimes” – “rarely” – “never/hardly ever”), as explained elsewhere (Velasco Garrido et al. 2018a).

### General health

Self-rated general health was addressed on a five-point Likert scale (“very good” – “good” – “fair” – “bad” – “very bad”) as recommended by WHO (de Bruin et al. 1996). For comparison purposes, health status was dichotomized merging the categories “very good” and “good” on the one side and “fair”, “bad” and “very bad” on the other side as usually done in health surveys (Subramanian et al. 2010). In addition, we asked a question referred to the issue of going to work despite feeling ill (presenteeism).

### Musculoskeletal pain

Musculoskeletal pain was assessed using the Subjective Health Complaints inventory (SHC) (Eriksen et al. 1999). The SHC consists of 29 ordinary somatic and psychological health problems and complaints organized in five subscales. For this paper we focus on the items of the “musculoskeletal pain” subscale (shoulder pain, neck pain, (upper) back pain, arm pain, lower back pain and leg pain). The participants are asked to rate the severity of each complaint during the past month (i.e., 4 weeks) in a four-point scale (“not at all” – “a little” – “some” – “serious”). According to the authors of the scale the inventory items can be dichotomized into “not at all” and “any” (including all other answer categories) (Eriksen et al. 1999). The single items might also be added into a score ranging from 0 to 18 for the musculoskeletal subscale, where 0 indicates no pain and 18 severe pain in all items.

### Statistical analysis

We did not perform any imputation for any variable, items left unanswered were treated as missing values. Descriptive statistics are reported as means with standard deviation (SD) for continuous variables, and as frequencies and percentages for categorical variables. We calculated two-tailed *p* values. Bivariate associations between type of job and musculoskeletal pain were explored with 2 × 2 contingency tables and Fisher’s exact test. Unadjusted odds ratios (OR) were calculated with binary logistic regression. Multivariate logistic regression was performed to explore associations between type of job and musculoskeletal pain (Model #1) and levels of exposure to physical demands and ergonomic challenges (climbing; overhead work; working with twisted upper body/forward flexion of the spine; handling tools and personal protection equipment; lifting/carrying heavy loads) and musculoskeletal pain (Model #2). Both models included the variables age and nationality. Differences in the musculoskeletal pain score were tested with the Mann–Whitney *U* test, since the variable had a non-normal distribution. The statistical significance level was set at *p* < 0.05. Statistical analyses were carried out using IBM® SPSS® Statistics (IBM Corp. released 2015. IBM SPSS Statistics for Windows, Version 23.0. Armonk, NY, USA). Graphs were edited using GraphPad Prism® version 8.2.1 for Windows (GraphPad Software, San Diego, California, USA).

## Results

**Fig. 1:**
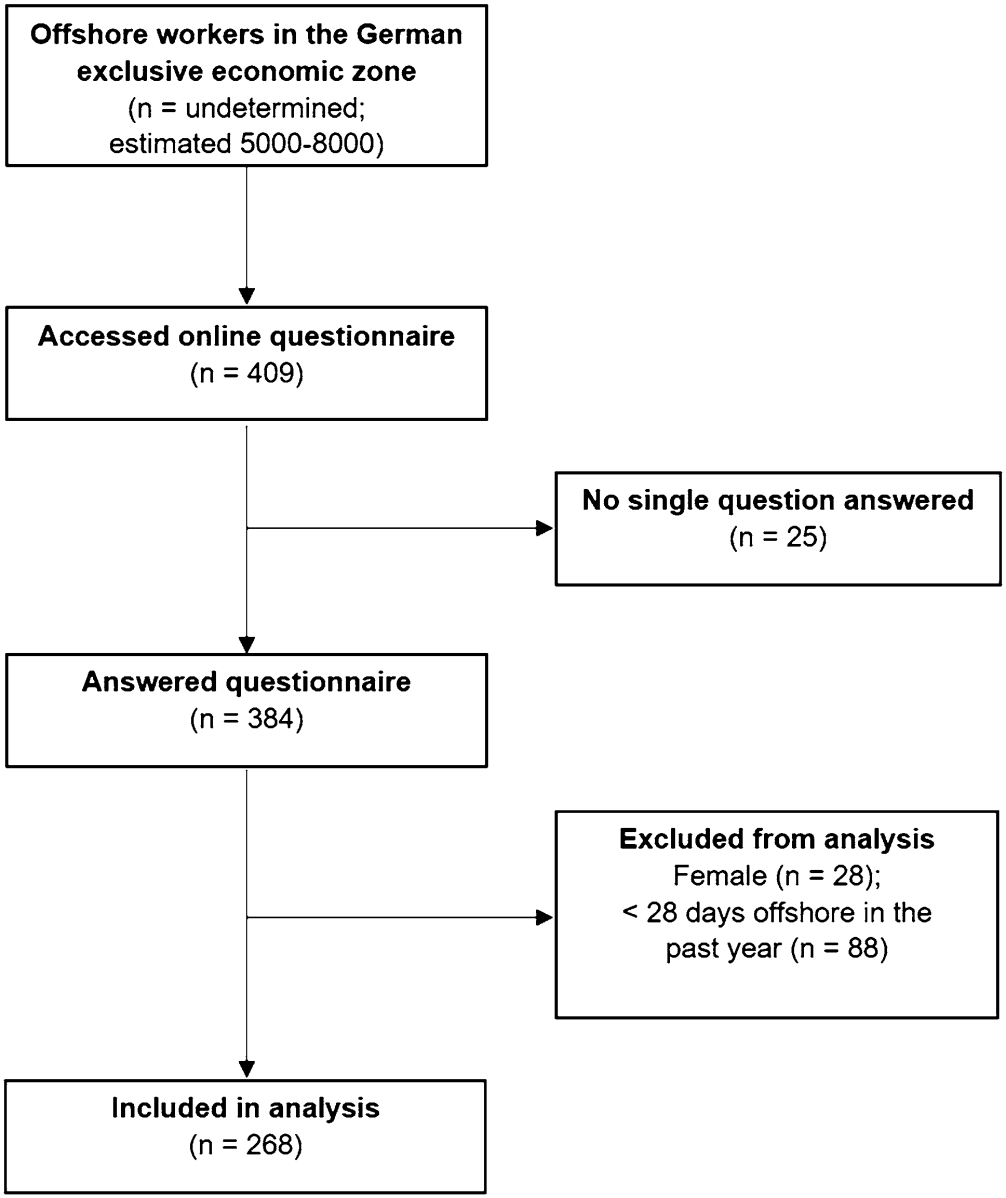
Selection of participants for analysis

The characteristics of the participants are presented in Table 1 and Fig. 1. The majority of the participants were younger than 50 years old (88.9%) and almost two thirds of them had more than 3 years of offshore experience. The sample mainly included technicians (*n* = 131), followed by managerial personnel including site/platform managers, quality managers, health-safety-environment managers (HSE-managers), supervisors (*n* = 110). Other occupations (*n* = 27) were paramedics, researchers and platform staff (i.e., catering, house-keeping).

**Table 1:** Characteristics of participants included in the analyses

Variable	*n*	%
Age (*n* = 268)		
20–34 years	116	43.3
35–49 years	122	45.5
≥ 50 years	30	11.2
Nationality (*n* = 262)		
German	234	89.3
Other	28	10.7
Offshore experience (*n* = 267)		
< 1 year	14	5.2
1–3 years	81	30.3
> 3 years	172	64.4
Type of job (*n* = 268)		
Technician	131	48.9
Other^a^	137	51.1
Work schedule (*n* = 268)		
Occasional deployments	35	13.1
Regular deployments	233	86.9
Of them with14/14 schedule^b^	198	84.5
Work shifts (*n* = 262)		
Day shifts only	130	49.6
Rotating shifts (day/night shifts)	132	50.4
Project phase (*n* = 267)		
Under construction	94	35.2
In operation	173	64.8

^a^“Other” includes site/platform managers, HSE-managers, quality managers and supervisors, paramedics, platform crew, research staff

^b^Working 14 days offshore followed by 14 days leave

### General health

Whereas 89.5% of respondents rated their health as “good” or “very good”, none of them reported having poor or very poor health. We found no statistically significant difference in the general health status between technicians and other occupations, neither in the bivariate (OR 0.64 95% CI 0.28–1.49) nor in the multivariate analysis adjusting for age and nationality (OR 0.60 95% CI 0.26–1.42).

Approximately one third (29.5%) of the workers had reported to have worked despite feeling ill instead of reporting sickness to the responsible person (i.e., paramedic, coordinator). The proportion was similar among technicians (30.8%) and the other occupations (28.2%), the small difference was not statistically significant (*p* = 0.752 in multivariate analysis adjusting for age and nationality).

### Musculoskeletal pain

**Fig. 2:**
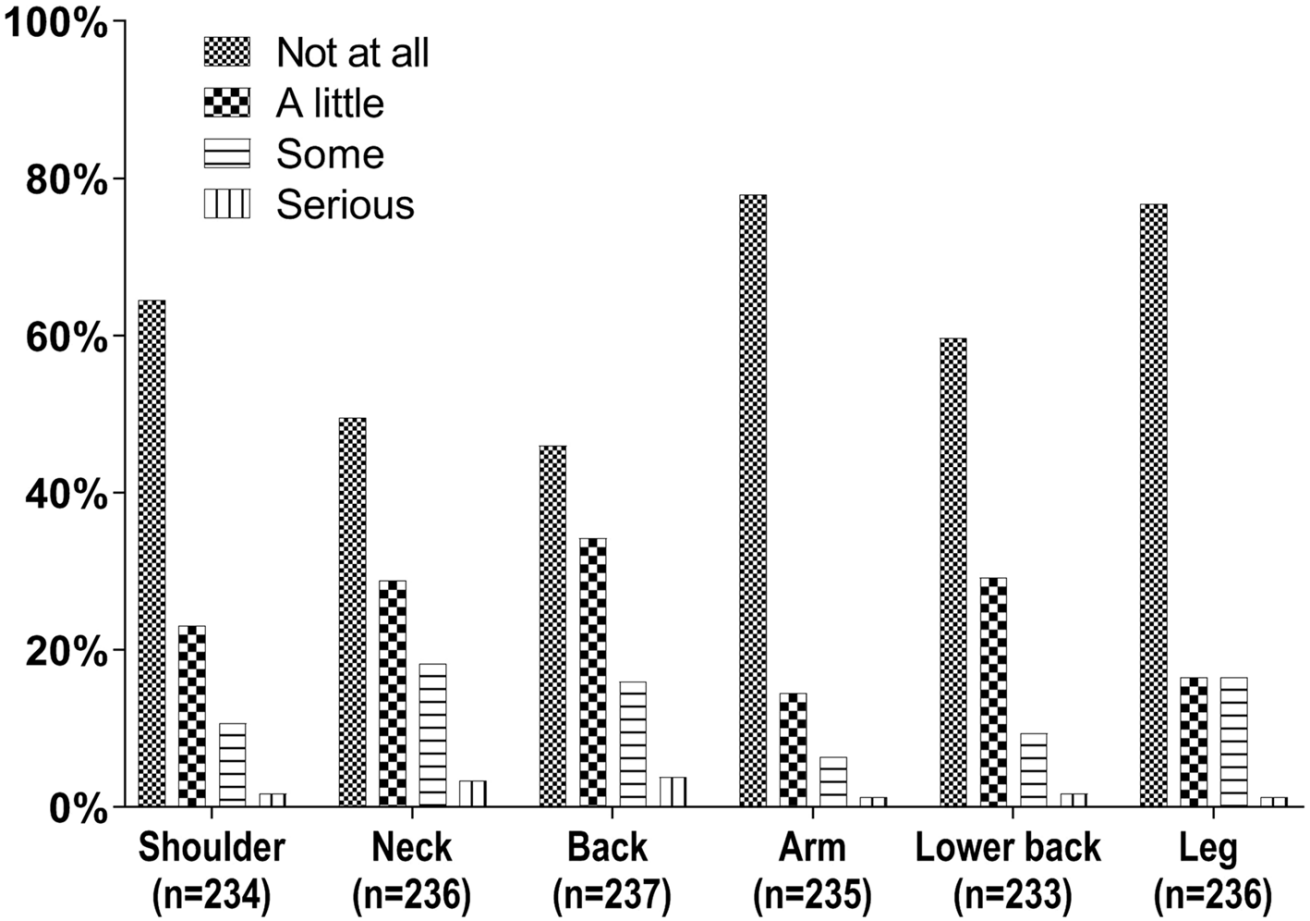
Frequency of musculoskeletal complaints among offshore wind workers by site

The most commonly reported localization was back pain with 54% of respondents reporting some level of pain followed by neck pain (50.4%), lower back pain (40.3%) and shoulder pain (35.5%). Leg pain was the least common with 22.1% of the sample reporting some level of pain in the past 30 days. Pain at more than two sites was reported by 38.6% of the participants. Pain at all six regions was reported by 6.6%. Figure 2 shows the distribution of the response categories for the different pain locations. Serious pain was not very prevalent in our sample. Serious pain was more frequent for the locations back with 3.8% of respondents and neck (3.4%). Serious pain was only half as frequent for the other regions with 1.7% of respondents for shoulder or lower back pain and 1.3% for arm or leg pain.

Technicians reported musculoskeletal pain more frequently than the other offshore occupations in all locations (Table 2). According to multivariate analysis, the strongest association was observed for arm pain (OR 3.13; 95% CI 1.58–6.19), followed by back pain (OR 1.97; 95% CI 1.15–3.39), with an OR > 1.00 indicating higher risk for technicians. The SHC-score for musculoskeletal pain was higher for technicians (mean 4.3; SD 3.6) than for other offshore workers (mean 3.3; SD 3.3), underlining the higher frequency and severity of musculoskeletal pain among technicians (mean score difference 0.96; 95% CI 0.04–1.87).

**Table 2:** Number and percentage reporting any musculoskeletal complaints by occupation

Item	Technicians *n* (%)	Other *n* (%)	Unadjusted^a^ OR (95% CI)	Adjusted^a,b^ OR (95% CI)
Neck pain	69 (59.5)	50 (41.7)	2.06 (1.22–3.45)	1.89 (1.11–3.22)
Back pain	74 (63.2)	54 (45.0)	2.10 (1.25–3.54)	1.97 (1.15–3.39)
Lower back pain	51 (44.3)	43 (36.4)	1.39 (0.82–2.35)	1.56 (0.90–2.70)
Shoulder pain	48 (42.1)	35 (29.2)	1.77 (1.03–3.04)	1.94 (1.11–3.40)
Arm pain	36 (31.0)	16 (13.4)	2.90 (1.50–5.59)	3.13 (1.58–6.19)
Leg pain	33 (28.4)	22.(18.3)	1.77 (0.96–3.27)	1.62 (0.86–3.03)

^a^Reference category “Other” (site/platform managers, HSE-managers, quality managers and supervisors, paramedics, platform crew, research staff)

^b^Adjusted for age and nationality

**Fig. 3:**
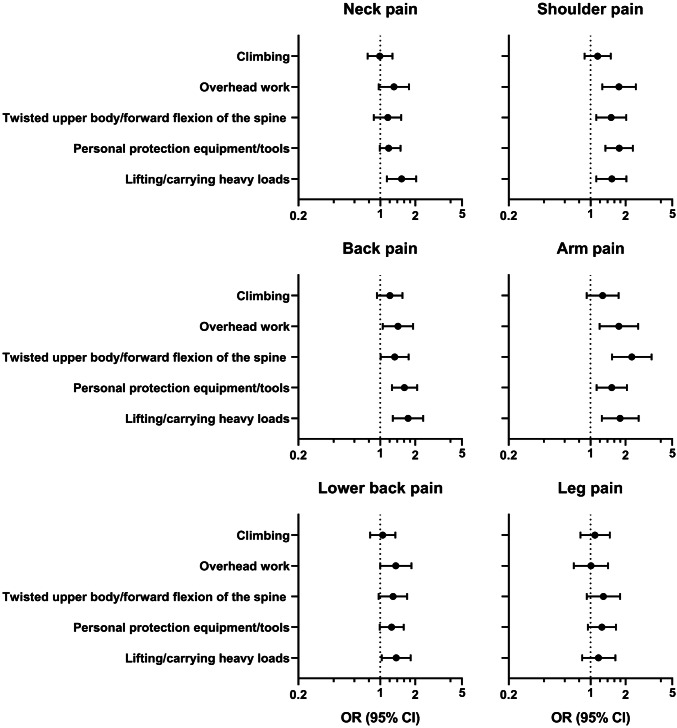
Ergonomic factors associated with musculoskeletal complaints at different locations of the body. Multivariate logistic regression, adjusted for age and nationality (*n* = 268)

Table 3 shows the distribution of physical demands among the different occupational groups. Figure 3 exhibits the results of multivariate analysis adjusted for age and nationality regarding the associations between several physical demands and ergonomic challenges with different localizations of pain. An OR > 1.00 indicates that with increasing exposure to the factor, the risk of reporting pain also increases. The strongest association was seen for working with twisted or bent upper body and arm pain (OR 2.26; 95% CI 1.53–3.33). Lifting and carrying heavy loads was statistically significant associated with all localizations of pain except for leg pain. Overhead work was strongly associated with arm pain (OR 1.75; 95% CI 1.20–2.56) and shoulder pain (OR 1.75; 95% CI 1.26–2.44). Handling and wearing heavy safety protection equipment and tools was associated with shoulder (OR 1.76; 95% CI 1.34–2.30), back (OR 1.61; 95% CI 1.26–2.07) and arm pain (OR 1.52; 95% CI 1.13–2.05). The associations between climbing and any of the pain localizations were weak and not statistically significant.

**Table 3:** Physical demands by occupation

Factor	Always		Often		Sometimes		Rarely		Never/hardly ever	
	*n*	%	*n*	%	*n*	%	*n*	%	*N*	%
Climbing (*n* = 254)										
Technicians	34	27.2	61	48.8	23	18.4	6	4.8	1	0.8
Other^a^	20	15.5	47	36.4	29	22.5	20	15.5	13	10.1
Overhead work (*n* = 254)										
Technicians	1	0.8	30	24.0	61	48.08	29	23.2	4	3.2
Other	0	0.0	14	10.9	46	35.7	41	31	28	21.7
Twisted upper body (*n* = 254)										
Technicians	5	4.0	57	45.6	42	33.6	18	14.4	3	2.4
Other	4	3.1	25	19.4	47	36.4	33	25.6	20	15.5
PPE/tools (*n* = 253)										
Technicians	30	24.0	44	35.2	28	22.4	20	16.0	3	2.4
Other	14	10.9	32	25.0	45	35.2	23	18.0	14	10.9
Lifting/carrying (*n* = 253)										
Technicians	9	7.2	58	46.4	44	35.2	12	9.6	2	1.6
Other	7	5.5	32	25.0	42	32.8	39	30.5	8	6.3

^a^“Other”: site/platform managers, HSE-managers, quality managers and supervisors, paramedics, platform crew, research staff

Regarding general working conditions (not shown in the figures), working in confined spaces was only associated with arm pain (OR 1.43; 95% CI 1.05–1.95). Restricted movement possibilities were associated with lower back pain (OR 1.31; 95% CI 1.01–1.70) but not with any other pain localizations.

## Discussion

To the best of our knowledge, our study is the first to address the issue of musculoskeletal pain among workers in the offshore wind energy industry. Due to the cross-sectional nature of our research as well as other limitations (see below), our findings need to be interpreted with caution and represent hypothesis to be confirmed with further research.

To date, musculoskeletal pain has been addressed among offshore oil and gas rig workers, indicating a high prevalence of this health disorder among this occupational group (Chen et al. 2005; Kalteh et al. 2018). However, although both workplaces share its geographical remoteness and hostile working conditions, working on oil and gas rig platforms is not the same as working in the offshore wind energy industry. Particularly, the work of offshore wind energy technicians is characterized by the almost daily transfer from vessels to the offshore wind energy installations, the climbing of ladders over several dozen meters, the carrying and handling of tools of up to 19 kg, as well as the wearing of heavy and bulky personal protection equipment of approximately 10 kg weight, and the frequent manual lifting of loads of up to 27 kg (Milligan et al. 2019). In our previous analysis of the data, we showed that offshore technicians regularly perform to overhead work, work in awkward postures, such as bent or twisted upper body, and lifting and/or carrying heavy loads (Velasco Garrido et al. 2018a). In the present analysis we found a relatively high prevalence of musculoskeletal pain among those surveyed. Compared to previous surveys with the SHC-instrument our respondents reported musculoskeletal pain more frequently than the general population (Eriksen et al. 1999) or than the working population (Indregard et al. 2013) although the workforce in offshore industry is relatively young.

It is already known that work-related musculoskeletal complaints are associated with occupational exposure to awkward postures and heavy lifting (da Costa et al. 2010). More specifically, there is evidence that overhead work and lifting loads are associated with the incidence of shoulder disorders (van der Molen et al. 2017). Indeed, we found statistically significant associations of overhead work not only with shoulder pain but also with arm and back pain. In our study, shoulder pain was additionally associated with heavy loads and working with twisted or bent upper body (awkward postures).

Regarding back pain, there is some evidence from the literature on occupations similar to wind technicians that ladder climbing and bending down is associated with lower back pain (Cooper et al. 2014). In addition, working with rotated or flexed/bent trunk has been associated not only with the incidence but also with recurrence of low-back pain in a prospective cohort study among the working population of The Netherlands (van den Heuvel et al. 2004). There is also strong evidence from longitudinal studies that frequent lifting and lifting loads over 25 kg are risk factors for the incidence of lower back pain (Coenen et al. 2014). In the present analysis, we did not find any statistically significant association between climbing and any of the pain localizations. This is surprising, since offshore wind industry workers describe climbing of ladders as being challenging, particularly when combined with carrying heavy tools and wearing safety clothing (i.e., survival suits) (Mette et al. 2017). Regarding working with bent/rotated trunk we found associations with shoulder, arm and back pain, but no statistically significant association with lower back pain. Similarly for carrying/wearing personal protection equipment and tools as well. Indeed, we only found a statistically significant association with lower back pain for lifting and carrying heavy loads. Due to the translation of the SHC inventory into German, our study may, however, have not been sensitive enough to reliably detect lower back pain as a separate entity, limiting the interpretability of our results. We cannot rule out that those reporting back pain were considering both their upper and lower back. This seems plausible, since the frequency of back pain in our study was clearly higher than that of lower back pain, although the opposite would be expected from the literature (Eriksen et al. 1999; Indregard et al. 2013). In summary, exposure to several different ergonomic challenges may explain at least to some extent the high prevalence of musculoskeletal pains of minor severity among offshore workers. This explanation is supported by our observation that technicians—who are exposed to combination of all above mentioned ergonomic factors more frequently and intensively (Velasco Garrido et al. 2018a)—reported musculoskeletal pain more frequently than other offshore occupations.

Besides the ergonomic factors discussed above, the incidence of musculoskeletal disorders (as shown by increased primary healthcare visits related to such problems) has also been associated with long term exposure to heavy physical work in general (Halonen et al. 2019). In a field study assessing cardiopulmonary parameters during preparatory trainings for offshore work, working in such installations has been demonstrated to imply heavy physical work (Preisser et al. 2019). Thus it is plausible that this factor could explain to some extent the high frequency of musculoskeletal pain among offshore workers observed in our present study.

In addition, it has been suggested that psychological factors such as perceived level of job stress and psychological demands also play an important role in the development of subjective health complaints, in particular musculoskeletal pain (Ihlbaek and Erkisen 2003; Oakman et al. 2017). Indeed, the prevalence of musculoskeletal pain has been associated with the specific occupational stressors of the offshore environment among Chinese oil rig workers (Chen et al. 2005). Other authors have suggested that suffering musculoskeletal pain may render workers less tolerant to the psychological demands of work (Bonzini et al. 2015). The interplay between psychological/psychosocial stress factors and musculoskeletal pain would be of particular relevance for the collective of offshore wind energy workers, for whom high levels of job related stress and psychological demands have been demonstrated (Mette et al. 2018a, b). Moreover, it has been suggested that there are reciprocal interactions between sleep quality and musculoskeletal pain, particularly multisite pain (Finan et al. 2013). These interactions might be relevant for offshore wind energy workers, since they frequently complaint about sleep disturbances as we have shown in this sample (Velasco Garrido et al. 2018b).

Interestingly, despite the relatively high prevalence of musculoskeletal pain, the respondents of our survey were in better subjective health than the general population with 89% reporting “good” or “very good” health as compared to 73% among German males (Robert Koch Institut 2014). The predominant pain was mainly of minor severity and has probably no major effect on general health feeling. The prevalence of “good” to “very good” self-rated health in our sample is comparable to that of academic professionals (92%) and substantially higher than that among manual labourers (76–82%) in the male German population (Burr et al. 2013). This finding probably reflects the selection of healthy workers according to the fitness requirements to work in offshore installations, which have been demonstrated to be more restrictive in Germany than in other European countries regarding some aspects such as cardiovascular fitness and respiratory health (Preisser et al. 2016).

Finally, compared to the general work-force in Germany with up to half of workers reporting having attended work when feeling ill in the past year (Hirsch et al. 2017), we found a lower level of presenteeism in our sample, although our questionnaire was not designed to address this problem in more detail. It has been discussed, that the difficulty in distinguishing between ‘home’ or leisure time and work, while offshore, as well as the often close relationships of the crew members, may promote sickness presenteeism (Krohne and Magnussen 2011). Although the problem seems to be lower than in the general work-force, it might be of relevancy since presenteeism can have adverse consequences for safety at work and for health and well-being in the long term (Sanderson and Cocker 2013; Skagen and Collins 2016).

### Limitations

Due to the lack of data on the workforce employed in the branch, we are not able to assess whether the respondents to our survey are representative. Although we cannot rule out, that the motivation to respond was triggered by suffering musculoskeletal pain, we do not think this is really an issue in our study since the information provided to promote the questionnaire was kept general mentioning “working and living conditions” and “physical and psychological burdens” as topics of the questionnaire. The introductory page of the questionnaire was also kept very general mentioning “working conditions, physical demands and psychological strains” topics.

The possibility of recall bias needs to be discussed. Recall bias may have affected the judgement of the respondents regarding their levels of exposure and the prevalence of musculoskeletal complaints. As explained in detail in our previous paper, we do not think that the observed tendency to report higher levels of exposure among those who answered the questionnaire, while on onshore leave would have biased the differences between technicians and non-technicians, since the proportion of workers responding to the questionnaire, while offshore was similar among both groups (42.7% among technicians, 43.1% among the other jobs) (Velasco Garrido et al. 2018a). Regarding musculoskeletal pain we did not observe any differences in the distribution of answers related to the time point of answering the questionnaire, thus we neither expect recall bias to affect these results.

The main limitation of our study is its cross-sectional design, which does not allow establishing temporality of cause-effect for the associations reported. However, the associations described in this paper are concordant with previous findings, as discussed above.

Another important limitation is given by the instrument we used to address physical strains. Self-assessment of physical strains is susceptible to the subjectivity of the worker and do not correlate well with measured exposures (Hansson et al. 2001). Objective and systematic assessment of ergonomic challenges can best be achieved using devices to measure musculoskeletal workload in the field. Field research was not under the scope of our study but future research should intend to quantify the strains reported by us. The CUELA system, for example, allows quantifying postural and kinetic workloads in complex work processes (Ellegast et al. 2009). Reliable measurements of strain from arm lifting, trunk bending, kneeling and squatting can be also achieved with small wireless devices (Korshøj et al. 2014; Hendriksen et al. 2020). Particularly small devices might be well compatible with the special protection equipment used in the offshore environment and could thus be suitable for future field research.

The SHC does not specifically address musculoskeletal pain but general health complaints. Unlike specific instruments for the study of musculoskeletal complaints—such as the Nordic Questionnaire on Musculoskeletal symptoms (Kuorinka et al. 1987)—the SHC does not include a body chart, limiting the interpretability and does not address other symptoms (i.e., movement limitations). Since the questionnaire used in our project was designed to address many aspects simultaneously (Velasco Garrido et al. 2018a, b; Mette et al. 2018a, b), the use of the SHC seemed more suitable than other more elaborated questionnaires for the analysis of musculoskeletal symptoms to avoid excessive length. Since we conducted an anonymous online survey, clinical assessment to detect underlying musculoskeletal disorders was not feasible. Thus no conclusions can be drawn regarding the clinical relevancy of pain of lower severity.

In summary, our paper is thus to be interpreted as hypothesis building and we see a need to address the relationship between work in offshore wind energy installations and the development of musculoskeletal pain and other complaints in further research with the aim of developing adequate and specific preventive strategies for this occupational group. To increase reliability, future studies should include objective measurements of ergonomic challenges—if feasible as field observation—as well as specific instruments for the assessment of musculoskeletal pain and other symptoms such as for example the Nordic questionnaires (Kuorinka et al. 1987).

Although the prevalence of serious pain at the time of the survey was not very high, we interpret the higher prevalence of at least some level of pain among technicians as an indicator of different preventive needs for this group. We, therefore, believe that our results indicate a need for tailored preventive interventions according to the tasks actually carried out by the offshore employees. Acknowledging that technical measures have priority when reducing physical demands, these may not always be implementable in the offshore environment. Thus we think, that ergonomic counselling could be integrated in the training that offshore wind energy workers have to undergo prior to their first offshore deployment as well as in the refresher-trainings. As part of such trainings, physiotherapists, for example, could provide practical demonstrations of compensatory exercises to prevent musculoskeletal complaints. The trainings in ergonomics should consider the special features of the workplace as well as the different settings and work situations (e.g., on deck, on the tower, cramped spaces). Another possibility would be to address ergonomic issues by occupational physicians within the fitness-checks that offshore workers regularly undergo. Furthermore, physical demands of single workers could be reduced by involving the workers in the allocation of tasks in such a way that physically demanding tasks are distributed evenly during the offshore deployments. For example in coordinating meetings, the workers could be given the possibility to opt for tasks that suit their individual performance and actual ability to work (Mette et al. 2019).

Finally, or results show that presenteeism matters in the offshore sector. Although offshore employees should report whether they feel able to work or not before any deployment and just before any upcoming transfer it happens that a considerable number of offshore workers want to work despite illness and do not report sickness. Thus, we think that the health competence of each individual employee should be strengthened, e.g., by emphasising personal responsibility when assessing warning signals from the body. In addition, chief officers should be made aware of their influence on their employees through their role model function and encourage their subordinates to take any symptoms of illness seriously. The “fitness-to-work” examination probably provides a suitable framework for enhancing awareness of offshore wind energy workers about this issue. In addition, contractual agreements should not penalize sickness-leave, since this may foster presenteeism (Kramer et al. 2013). In our view, the problem of presenteeism should be considered in all organisations’ HSE policies.

## Conclusions

Workers in offshore wind energy installations report frequent non-severe musculoskeletal pain. Offshore wind energy technicians are particularly at risk of suffering from arm, shoulder, neck and back pain compared to workers performing other tasks at the offshore wind sites. Ergonomic challenges such as overhead work, handling heavy tools and wearing heavy protection equipment are factors associated with musculoskeletal complaints. Occupational health counselling of offshore wind energy workers needs to consider the specific tasks of the employee and address ergonomic issues. According to our results, health promotion and preventive interventions should be tailored to the occupational subgroup of technicians, since they show higher levels of musculoskeletal complaints, which may result—at least partly—from their specific job demands.
